# The influence mechanism of upward social comparison on work withdrawal behavior of rural-origin civil servants: based on the mediating effect of ego depletion and the moderating effect of social mobility belief

**DOI:** 10.3389/fpsyg.2025.1653112

**Published:** 2025-09-01

**Authors:** Jingyuan Zhang, Fengjuan Yan, Lin Meng

**Affiliations:** ^1^School of Business Administration, Shandong Women's University, Jinan, China; ^2^School of Public Administration, Shandong Normal University, Jinan, China

**Keywords:** upward social comparison, work withdrawal behavior, ego-depletion, social mobility belief, rural-origin civil servants

## Abstract

As the sustained popularity of civil service careers persists, a significant number of young people from rural backgrounds have entered the civil service. However, due to differences in social status and background, rural-origin civil servants are prone to engaging in upward social comparison. This results in ego depletion and subsequently induces work withdrawal behavior, adversely affecting team harmony and sustainable development. This study empirically examines the mechanism by which upward social comparison influences work withdrawal behavior among rural-origin civil servants. The concepts of ego depletion and social mobility belief are introduced, with ego depletion further categorized into three dimensions: cognitive, emotional, and behavioral dimensions. Analysis of questionnaire data collected from 1,137 rural-origin civil servants in Jinan, Shandong Province, yielded the following findings: Upward social comparison significantly predicted work withdrawal behavior. Cognitive and emotional ego depletion positively mediated the relationship between upward social comparison and work withdrawal behavior. Furthermore, the mediating effect of emotional ego depletion was stronger than that of cognitive ego depletion. Social mobility belief negatively moderated the pathways through which upward social comparison influences cognitive ego depletion, behavioral ego depletion, and work withdrawal behavior. This research offers a new perspective for understanding the psychological mechanisms underlying work withdrawal behavior among rural-origin civil servants and provides a theoretical basis for targeted interventions.

## 1 Introduction

In recent years, the phenomenon of “civil service fever” in China has intensified amid growing labor market competition and the ongoing refinement of the civil service examination and appointment system. Through competitive selection examinations, numerous individuals from rural backgrounds have been recruited into provincial and municipal civil service agencies, thereby gaining entry into the civil service.

It should be emphasized that the “rural-origin civil servants” group central to this study, while primarily defined by the household registration system, exhibits considerable complexity in its connotations owing to social mobility and urbanization in contemporary China. Although household registration status has historically served as an institutional marker crucial for distinguishing urban–rural identities and accessing social resources, it may inadequately capture individuals' actual experiences regarding identity formation, cultural adaptation, and socio-psychological integration. Many civil servants holding rural household registrations may have resided in urban areas for higher education or employment years prior to passing their examinations; conversely, newly recruited rural-origin civil servants might experience significant cultural transitions. Factors including the duration of urban residence, the extent of lifestyle change, psychological detachment from original rural communities, and internalization of urban professional identities and values can profoundly influence subjective perceptions of “rural identity” and sensitivity to social comparisons. In other words, “rural origin” represents not merely a static administrative label but a dynamic, multi-dimensional socio-psychological construct. Reliance solely on household registration to define the study population risks overlooking heterogeneity arising from diverse urbanization experiences, potentially compromising the precision and generalizability of findings. Consequently, while household registration status serves as the primary screening criterion in this study's operationalization of “rural-origin civil servants,” the limitations inherent in this conceptualization are acknowledged. Subsequent analyses will incorporate key control variables such as the length of urban residence. Furthermore, the potential moderating effects of urbanization experiences on identity and psychological mechanisms will be rigorously considered in the discussion. This approach aims to provide a more comprehensive understanding of the “identity predicament” faced by this group and its implications for work behavior. Rural background is defined by three criteria: possessing agricultural household registration status at birth, which is currently retained even if the individual has subsequently relocated to urban areas; primary residence during elementary school years being located in a township or village; and at least one parent having engaged long-term in agricultural labor or non-agricultural work within rural areas.

Although individuals from rural backgrounds successfully enter civil service positions, perceived differences in social status exist due to objective disparities in social resources—such as income, education, and position—associated with distinct social identity groups (Kraus et al., 2009, 2012). The social origin of rural-born civil servants, acting as an enduring cultural imprint, profoundly influences their cognition and behavior (Martin et al., 2016; Sharps and Anderson, 2021). Furthermore, the combination of vertical delegation and horizontal competition within the civil service system induces psychological distress, which manifests as discomfort and maladjustment among officials of rural origin due to disillusionment and anxiety (Song et al., 2021). This may lead to inaccurate evaluations of work performance, both their own and others', as factors including organizational systems, self-perception discrepancies, and strained interpersonal relationships compound (Phuong et al., 2018). In addition, under the dual political and economic structure of urban and rural areas in China, there is a gap in the economic and living conditions of rural-origin civil servants compared to non-rural civil servants. This gap hinders their effectiveness and status recognition (Zhang et al., 2023); it leads to a lack of belonging to the organization, and makes them prone to adverse mentalities such as jealousy and anxiety (Cai et al., 2022), which contribute to work withdrawal behavior among rural-origin civil servants. Work withdrawal behavior is not only detrimental to personal mental health and work performance but also hinders the smooth progress of departmental work (Zhang et al., 2023). Therefore, it is of great practical significance to study the work withdrawal behavior of rural-origin civil servants.

Work withdrawal behavior is a series of negative work behaviors adopted by individuals to avoid work or weaken the socio-psychological connection between themselves and the organization (Aggarwal et al., 2020; Xu et al., 2022). Work withdrawal behavior is covert and retaliatory, which has the potential to negatively affect individual and organizational performance and development (Zimmerman and Darnold, 2009; Serfraz et al., 2022). At present, academic research on the influencing factors of work withdrawal behavior is concentrated in the following aspects. First, at the individual level, researchers analyze the reasons for work withdrawal behavior from the perspective of personal traits. Scholars believe that work withdrawal behavior is affected by factors such as individual emotional stability (Lebreton et al., 2004), degree of emotional labor (Gyu-Min et al., 2016), surface acting behavior (Scott and Barnes, 2011), subjective will (Bowers et al., 2022), and self-monitoring level (Bowers et al., 2022). Secondly, at the organizational environment level, work withdrawal behavior is affected by imperfect performance appraisal standards (Coutinho et al., 2018), lack of fair and effective communication, a safe organizational atmosphere (Tsai et al., 2015), an imperfect employee behavior supervision system, a mismatched reward and punishment system (Chen, 2020), lack of competitiveness and transparency of salary, unreasonable task allocation (Lambert et al., 2023), and lack of timely relief from multiple sources of organizational pressure (Yu and Wang, 2022). Moreover, research has found that when there is obvious favoritism or serious nepotism in the organization, individuals are more likely to lose loyalty to and trust in the organization or superiors, stimulate their cynicism, and exhibit increased work withdrawal behaviors (Abubakar et al., 2017). Third, at the leadership level, leadership style is a critical perspective to explore. Specifically, research shows that positive leadership behaviors, such as transformational leadership and supervisor support, reduce work withdrawal behaviors (Elshaer et al., 2022); conversely, abusive management and other negative leadership behaviors increase work withdrawal behavior (Offergelt and Venz, 2023). Compared with the organizational and leadership levels, research focusing on the individual level, especially the psychological level, of rural-origin civil servants is more practically effective in preventing and intervening in their work withdrawal behavior.

Social comparison is a universal psychological phenomenon (Wrede et al., 2021). Individuals in different environments often unconsciously develop a tendency toward social comparison. According to different objects of comparison, social comparison is divided into parallel social comparison, upward social comparison, and downward social comparison (Bella, 2024). There are two consequences of social comparison: If an individual perceives themselves to be in a different state from the upward comparison target, a contrast effect is generated, leading to a sense of inferiority and more negative self-evaluation (Corcoran et al., 2020). Conversely, if individuals expect that they will be in the same state as the upward comparison target, the assimilation effect will occur, and their sense of self-worth will be enhanced (Zell and Strickhouser, 2020). According to social comparison theory, people have a drive to hope that they are better than others, which leads to upward social comparison (Rehman Khan, 2021). Upward social comparison refers to the behavioral tendency of individuals to compare themselves with others who have advantages in the process of social comparison. Individuals measure their own value by comparing themselves with those who perform better or achieve more (Li and Wang, 2023). The more frequently an individual makes upward social comparisons, the more severely their self-efficacy is reduced. According to the contrast effect theory, when individuals use outstanding others as reference points for comparison, they feel threatened due to the gap between themselves and others. At the same time, they lower their self-evaluation (Zhao et al., 2022), which leads to negative emotional reactions (Aldabbas and Bettayeb, 2024). According to social information processing theory, the information in the external environment affects the attitudes and behaviors of employees (Harter et al., 2020). Frequent upward social comparison makes rural-origin civil servants feel the psychological disadvantage and self-threat of “I am not as good as others in everything”, which reduces their level of self-esteem (Panagopoulos et al., 2016) and increases their sense of loneliness (Arnold et al., 2021). In the long run, rural-origin civil servants are prone to anxiety and doubt about their own identity, tend to think their efforts are futile, and lose motivation for work. A series of work withdrawal behaviors manifest, such as being late, absent, taking long breaks, fabricating excuses, and dispersing work energy in the workplace. In view of this, this study attempts to discuss the relationship between upward social comparison and work withdrawal behavior among rural-origin civil servants. Therefore, this study applies upward social comparison theory to rural-origin civil servants operating within China's urban–rural dichotomy. This group faces not only generalized social comparison pressures but also profound identity marking alongside potential institutional and cultural disparities. Examining how upward social comparison triggers work withdrawal behavior within this unique context can enrich the application boundaries of social comparison theory within organizational behavior, particularly in public sector organizational behavior.

Negative emotions such as jealousy, feelings of inferiority, anxiety, and depression caused by upward social comparison can easily lead to individual ego depletion (Ming et al., 2020). The ego depletion theory points out that an individual's self-control resources are limited over a certain period of time; negative life experiences or pressure, as well as self-control demands, will consume these resources (Moayery, 2021). The state of depleted self-control resources is referred to as ego depletion. When individuals are in a state of ego depletion, psychological exhaustion occurs, and the likelihood of externalizing (such as aggressive and addictive behaviors) and internalizing (such as anxiety and depression) problems increases (Wrede et al., 2021). Ego depletion is an important reason for individual work withdrawal behavior, and the negative emotions generated by the upward social comparison of individuals force them to engage in forced control (Englert and Bertrams, 2013). Self-control is divided into autonomous control and forced control by self-determination theory. Autonomous control refers to complete control by oneself or the real self, reflecting an individual's ability to do things that are interesting, important, or at their own discretion; forced control refers to control that is rarely determined by oneself, which carries a sense of pressure, compulsion, or deception. Some studies have found that autonomous control does not cause ego depletion, while forced control can lead to ego depletion (Liu and Ma, 2020).

Although ego depletion theory provides a critical perspective for understanding willpower depletion and behavioral control failures and is widely applied in organizational behavior research, the robustness and replicability of its core assumption—that self-control relies on a finite, depletable general resource—have faced empirical challenges in recent years. These challenges primarily concern the nature of the proposed “resource,” the universality of depletion effects, and the ecological validity of experimental paradigms. However, within the specific context examined in this study—the sustained psychological pressure experienced by rural-origin civil servants due to upward social comparisons—the concept of ego depletion retains considerable theoretical validity and explanatory advantage, particularly when operationalized across cognitive, emotional, and behavioral dimensions. Civil service roles demand high levels of emotional labor, continuous cognitive monitoring, and behavioral self-discipline. When engaging in upward social comparisons, rural-origin civil servants experience perceived status disadvantage, resource disparity, and potential injustice, which persistently trigger cognitive reappraisal, emotional regulation, and behavioral inhibition. This sustained, multi-dimensional demand for self-regulation constitutes the core characteristic of the “depletion” process. Even if the underlying mechanisms may involve shifts in motivation or belief, the subjective experience and observable behavioral manifestations align with the classic description of “resource” consumption leading to diminished subsequent regulatory capacity. Furthermore, differentiating ego depletion into cognitive, emotional, and behavioral components directly addresses critics' concerns regarding the excessive vagueness of the “general resource” concept. This differentiation allows for a more precise capture of the differential impact of upward social comparisons across distinct functional domains, rather than relying on an aggregate depletion score. Consequently, it facilitates the identification of specific pathways through which depletion occurs, enhancing the theory's precision and predictive power.

It is important to note that ego depletion specifically concerns the depletion of internal psychological resources—cognitive resources, emotional regulation resources, and behavioral control resources—resulting in diminished self-control capacity. Work withdrawal behavior, in contrast, represents intentional actions involving work avoidance or organizational disengagement, potentially stemming from psychological states such as ego depletion. Ego depletion constitutes an antecedent state of reduced energy availability, while work withdrawal behavior constitutes a consequential action characterized by deliberate reduction or cessation of work effort. These constructs are distinct in conceptual definition and operational measurement.

As an intrinsic motivation factor, social mobility belief is an expectation for individuals to achieve upward social mobility and has motivational effects on individual attitudes and behaviors. To achieve upward mobility to a higher social level, an individual's goal orientation and pursuit can be regulated by upward social mobility (Liu and Ma, 2020). Empirical research also shows that social mobility belief can affect individual motivation to pursue success (Han et al., 2023), choice of goal orientation (Dhoore et al., 2019), individual effort, and behavior (Romme, 2021). Social mobility belief can improve individual confidence by reducing the cost of information acquisition, providing psychological counseling channels, and coordinating interpersonal cooperation. Within China's civil service system, promotion and career advancement represent key motivating factors. For rural-origin civil servants, social mobility belief is linked not only to enhanced personal economic status but also profoundly to the consolidation of identity following departure from agricultural life and the potential for intra-system stratification mobility. Consequently, the strength of this belief holds particular theoretical significance for regulating upward comparison pressure arising from status differences and its subsequent behavioral outcomes. Examining this regulatory mechanism connects perceptions of macro-level social structures with micro-level individual psychological processes and organizational behaviors, providing a novel perspective for understanding how social structures influence internal organizational conduct through individual beliefs.

The existing literature has enriched the research field of work withdrawal behavior among civil servants, providing theoretical support for this study. However, there is room for expansion: there is little literature on the relationship between upward social comparison, ego depletion, and work withdrawal behavior. The pairwise studies on the three variables are mostly focused on college students, adolescents, social network use, and other groups or backgrounds, with few studies on civil servants. The working status and mental health of civil servants are related to the administrative efficiency of the government and affect the implementation of national policies. However, in the previous research literature, there are few studies on the mental health status and related interventions for rural-origin civil servants. Based on this, rural-origin civil servants are taken as the research object of this article, as they are more likely to trigger upward social comparison behavior within the civil servant team. Through data analysis in Jinan City, Shandong Province, this study aims to explore the correlation between upward social comparison, ego depletion, and work withdrawal behavior among rural-origin civil servants, as well as the action mechanism of social mobility belief among the three variables. This is intended to provide practical methods and a basis for intervention research to improve the mental health level of civil servants, especially rural-origin civil servants, in the future.

## 2 Theoretical analysis and research hypothesis

### 2.1 Direct effect of upward social comparison on work withdrawal behavior

Upward social comparison refers to the strong internal drive that individuals generate when evaluating their current situation and environment in situations where objective evaluation criteria are lacking (Uziel and Cohen, 2020). They compare themselves with those around them who are doing better, to engage in self-confirmation behavior. According to threat effect theory (Ghosh and Irum, 2023), when individuals compare themselves with those who are better off, this threatens their self-esteem, resulting in a sense of deprivation (Pan and Zhao, 2024). Previous studies have shown that upward social comparison can have a series of effects on rural-origin civil servants, such as causing anxiety, jealousy, depression, and reducing individual happiness (Zheng et al., 2020; Lewis, 2021). It can be seen that upward social comparison may closely relate to the generation of negative work behaviors in organizational contexts.

Work withdrawal behavior is a typical negative work behavior that employees engage in to avoid work tasks or situations (Peng and Li, 2023). Work withdrawal has two dimensions: psychological withdrawal and behavioral withdrawal (Lehman and Simpson, 1992). Specifically, psychological withdrawal refers to employees withdrawing from the work situation in psychological activities, including chatting about private topics during working hours, mind wandering during working hours, and developing a tendency to quit. Behavioral withdrawal refers to employees' withdrawal from work in actual behavior, such as tardiness, absenteeism, and napping at work. Through upward social comparison, rural-origin civil servants feel jealous or unhappy when they perceive the gap in social and economic status, personal performance, work achievements, and personal image. To alleviate and cope with this negative emotional experience, rural-origin civil servants generate corresponding behavioral responses or action strategies, such as avoiding the comparison object and the corresponding comparison environment (Zhang et al., 2020; Gaviria et al., 2021). Therefore, rural-origin civil servants develop an evasive mentality after making upward social comparisons. For the purpose of escape, rural-origin civil servants tend to choose strategies to reduce direct interactions with the comparison object (colleagues, leaders) and the comparison environment (workplace). In the work environment, they often exhibit absenteeism, a tendency to turnover, and other work withdrawal behaviors (Wu et al., 2021). Therefore, this study proposes the following hypothesis:

**H1:** Upward social comparison among rural-origin civil servants has a significantly positive effect on their work withdrawal behavior.

### 2.2 Direct effect of upward social comparison on ego depletion

When there is competition and uncertainty in the environment, individuals are more inclined to compare themselves with others to evaluate themselves (Campbell et al., 2017). When individuals recognize and evaluate themselves through external information, they will change their perception and evaluation, blurring the clarity of self-concept. As a group with established roles, the social status of civil servants is restricted by various systems, and their economic status cannot catch up with that of high-income people. Therefore, the authority of civil servants' professional status is constantly declining, which reduces their sense of identity regarding their professional status (Frogeli et al., 2022). According to the self-control resource model, self-control activities such as attention control, emotional regulation, and cognitive processing consume limited resources. The consumption of these resources can impair the ability to engage in self-control tasks. When this impairment occurs, ego depletion will follow (Fan W. et al., 2023). The process by which individuals compare their circumstances (including abilities, perspectives, physical health, etc.) with those of others involves distinct components: cognition, emotion, and behavior. Research has found that when making upward social comparisons, self-evaluation is reduced, and negative emotions are generated (Hui et al., 2022). The stronger the negative emotions produced, the higher the degree of fatigue. Due to upward social comparison, rural-origin civil servants are prone to experiencing contrast effects, resulting in psychological imbalances in the gap between themselves and others. Negative emotions such as anxiety and depression are produced (Neff et al., 2015). Studies have shown that negative emotions, such as anxiety, can lead to ego depletion. According to the theory of limited self-control resources, previous energy consumption reduces performance in subsequent self-control tasks (Vohs et al., 2008).

Specifically, within the rural-origin civil servant population: cognitively, processing comparative information, suppressing downward comparison impulses, and maintaining or reconstructing impaired self-concepts demand significant cognitive control resources (Wu et al., 2023). This results in self-cognitive depletion, manifesting as self-doubt, diminished self-worth, and pessimistic future outlooks. Affectively, both the immediate suppression of negative emotions arising during comparison and the sustained experience of unresolved negative emotions consume substantial emotional regulation resources, leading to self-emotional depletion. Behaviorally, inhibiting direct reactions prompted by cognitive biases and emotional stress, along with sustaining role-appropriate positive behaviors such as dedication and self-discipline, requires considerable behavioral regulation resources (Matin et al., 2024), potentially causing self-behavioral depletion. Based on this, this study proposes the following hypotheses:

**H2:** Upward social comparison has a significant positive effect on ego depletion;

**H2a:** Upward social comparison has a significant positive effect on ego-cognitive depletion;

**H2b:** Upward social comparison has a significant positive effect on ego-emotional depletion;

**H2c:** Upward social comparison has a significant positive effect on ego-behavioral depletion.

### 2.3 Direct effect of ego depletion on work withdrawal behavior

It is found that employees engage in work withdrawal behavior because they intentionally avoid work or want to weaken their psychological connection with the organization. Employees perceive that something in the organizational environment causes them to experience negative emotions. To avoid these emotions, they adopt a withdrawal in attitude and behavior. In addition, work withdrawal behavior follows a sequence in time. First, it manifests as slight withdrawal behavior, which then gradually evolves into late arrival, early departure, absence from work, and finally resignation. The intensity of withdrawal increases from the initial behavior. According to ego depletion theory, the resources possessed by an individual are limited, and self-control consumes these limited resources. Resource depletion manifests across three dimensions. Cognitive resource depletion induces shallow cognitive processing and cognitive biases, characterized by underestimated self-capability and pessimistic future outlooks (Leahy and Sweller, 2019). Such negative cognition frequently triggers job dissatisfaction and work avoidance tendencies, including psychological withdrawal and turnover intention. Emotional resource depletion impairs the ability to manage negative affect and sustain work engagement (Van Bruggen et al., 2025). Persistent negative emotions such as frustration and burnout directly drive physical or psychological disengagement from work, evidenced through absenteeism, tardiness, early departure, and reduced attentiveness. Behavioral control resource depletion compromises impulse inhibition and normative compliance capacities (Xu et al., 2024), commonly precipitating impulsive decisions to evade work responsibilities or weaken organizational bonds, manifested through diminished effort exertion, excuse-making, and task avoidance.

According to social exchange theory, when one party provides the resources needed by the other, the other party will give back. But when one party's resources are damaged, it may strike a balance by retaliating against the other. The theory also states that there is a two-way contractual relationship between individuals and organizations. When rural-origin civil servants are unable to perceive fair salary benefits, work opportunities, and respect and recognition from the organization through upward social comparison, they are less willing to repay the organization, resulting in work withdrawal behavior. When an individual is in an environment that can meet his needs, his internal motivation will be enhanced. The degree of satisfaction of the basic psychological needs of individuals will be affected by the external environment and will show different attitudes and behaviors. From the perspective of the three dimensions of ego depletion: ego-cognitive depletion occurs when rural-origin civil servants perceive no realization of self-worth in the organization, leading to cognitive depletion. They will reduce their internal motivation to return to the organization and exhibit withdrawal behavior. Ego-emotional depletion and ego-behavioral depletion mean that when rural-origin civil servants feel their work cannot meet their psychological needs and cannot develop a sense of belonging and responsibility, this will stimulate negative attitudes such as burnout and laziness, and reduce their enthusiasm for work. To make up for the “gap” caused by resource depletion, self-control ability will be weakened, leading to impulsive behaviors (Hagger et al., 2019). According to scarcity theory, if individuals are in a state of deficiency, they tend to pay attention to what they lack. The cognitive load brought by this attention ultimately leads to their neglect of the future, resulting in unfavorable decisions characterized by short-sighted behavior (Mani et al., 2013), which can easily lead to work withdrawal. Based on the above analysis, this study proposes the following hypotheses:

**H3:** Ego depletion has a significant positive effect on work withdrawal behavior;

**H3a:** Ego-cognitive depletion has a significantly negative effect on work withdrawal behavior;

**H3b:** Ego-emotional depletion has a significant positive effect on work withdrawal behavior;

**H3c:** Ego-behavioral depletion has a significant positive effect on work withdrawal behavior.

### 2.4 Mediating effect of ego depletion

Ego depletion refers to the temporary reduction of an individual's ability or willingness to participate in subsequent volitional activities as a result of their participation in previous ones (Martijn et al., 2007). According to the self-control resource model, the consumption of mental resources in early volitional activities leads to ego depletion, which includes controlling the environment, controlling oneself, making choices, and initiating actions. The social background, as a cultural imprint, is something that rural-origin civil servants cannot eliminate and has a profound effect on their cognition and behavior (Hunter et al., 2020). Upward social comparison, as a more complex volitional activity, involves the digestion or adjustment of individual emotions and cognition. A significant amount of individual psychological resources is consumed in the process of upward social comparison. Simultaneously, ego depletion is categorized into ego-cognitive depletion, ego-emotional depletion, and ego-behavioral depletion. Ego-cognitive depletion refers to the subjective perception that an individual is in an inferior position relatively. After the consumption of cognitive resources, an individual may hold a negative attitude toward work (Hunter et al., 2020), including doubts about personal ability, jealousy of other people's achievements, and a reduction of self-identity. Ego-emotional depletion mainly refers to the consumption caused by personal emotions that make it difficult for the body and mind to maintain a normal state, accompanied by jealousy, anxiety, depression, frustration, and other psychological feelings (Konze et al., 2019). Ego-behavioral depletion refers to impulsive behavior manifested by the weakening of self-control ability after the consumption of self-control resources, such as substance abuse, excessive consumption, binge eating, aggressive behavior, and so on (Mackey et al., 2020; Li et al., 2022).

This study posits that upward social comparison among rural-origin civil servants constitutes a process highly demanding of psychological resources (Saleem et al., 2022). Cognitively, processing comparison information, suppressing impulses for downward comparison, and maintaining or reconstructing impaired self-concepts consume considerable cognitive control resources (Friese et al., 2019). This leads to ego-cognitive depletion, manifesting as self-doubt, diminished self-worth, and future pessimism. Emotionally, whether suppressing immediate negative emotions arising from comparison to comply with workplace norms or persistently experiencing these unresolved negative emotions, significant emotional regulation resources are expended (Thai et al., 2022). This results in ego-emotional depletion, characterized by emotional exhaustion, irritability, and impaired emotion regulation. Behaviorally, suppressing direct behavioral responses stemming from cognitive biases and emotional pressure, alongside sustained maintenance of positive behaviors required by the civil servant role, demands substantial behavioral regulation resources (Chen F. et al., 2024). This contributes to ego-behavioral depletion, evident in weakened impulse control and reduced behavioral self-discipline. The civil servant role necessitates continuous cognitive monitoring, emotional labor, and behavioral regulation (Ford and Gross, 2018). Upward social comparison threat significantly imposes additional demands on these three regulatory domains, with ego depletion effects further compounded by the chronic nature of these regulatory requirements.

Furthermore, resource conservation theory indicates that individuals in a state of resource depletion tend to engage in behaviors aimed at protecting their remaining resources. Work withdrawal behaviors, such as tardiness, leaving early, reduced effort, and psychological detachment, represent a typical resource conservation strategy. These behaviors seek to prevent further depletion or facilitate immediate recovery by reducing work investment. Consequently, the ego depletion triggered by upward social comparison, particularly the exhaustion of cognitive and emotional resources, serves as the key psychological mechanism predicting subsequent work withdrawal behaviors. Therefore, this study proposes the following hypotheses:

**H4:** Ego depletion plays a positive mediating effect in the influence of upward social comparison on work withdrawal behavior;

**H4a:** Ego-cognitive depletion has a positive mediating effect between upward social comparison and work withdrawal behavior;

**H4b:** Ego-emotional depletion has a positive mediating effect between upward social comparison and work withdrawal behavior;

**H4c:** Ego-behavioral depletion has a positive mediating effect between upward social comparison and work withdrawal behavior.

### 2.5 Moderating effect of social mobility belief

Social mobility is the basis and guarantee for the stable and sustainable development of a country and is also an important means of achieving social equity. Social mobility is divided into objective and subjective aspects (Iversen et al., 2019). Objective social mobility refers to the rise or fall of social and economic status, essentially known as social class mobility (Sepulveda, 2023). Social mobility belief is the subjective perception or judgment that objective social mobility maps to the individual, representing people's subjective judgment and expectation of the possibility of upward (or downward) change in their social class or socioeconomic status (Seto, 2024). Social mobility belief encompasses two dimensions. One is the perception and belief about the possibility of changing socioeconomic status, such as the extent to which a society allows its members to achieve better economic status through individual actions like effort (Yoon and Kim, 2018). In other words, individuals believe that everyone has the potential to move toward the upper echelons of society (Davidai and Gilovich, 2015). The second dimension is the expectation and belief in personal social mobility, which refers to the extent to which one believes that they can improve their future social and economic status through effort and hard work (Eo and Kim, 2020). Current studies combine these two dimensions to reflect the overall level of individual social mobility beliefs. According to expectancy-value theory, the strength of behavioral motivation is determined by individuals' expectations of success and their value assessments of outcomes. A strong social mobility belief indicates high expectations that effort leads to status improvement.

Based on social comparison theory, whether upward comparisons produce threat or motivation depends on an individual's assessment of the attainability of the comparison target's status (Caliskan et al., 2024). Rural-origin civil servants possessing strong beliefs in social mobility are more likely to perceive high-performing non-rural colleagues as attainable goals rather than insurmountable gaps. This positive interpretation directly mitigates the negative impacts of upward comparisons on self-concept and emotional wellbeing (Zhang et al., 2025). This indicates that the negative emotions experienced by rural-origin civil servants, such as inferiority and jealousy caused by socioeconomic status, working environment, and comparison objects, can be alleviated by the belief in social mobility. Furthermore, self-determination theory states that compulsive control consumes energy and vitality, while autonomous control does not. The belief in social mobility can promote individual self-control and reduce energy consumption; that is, the forced control caused by ego depletion is transformed into autonomous control. A high belief in social mobility represents a significant psychological resource. It enhances individuals' perceived control over the future and fosters optimistic expectations while providing meaning and purpose for coping with comparative pressures and identity dilemmas (Sagioglou et al., 2019). This facilitates the mobilization of resources to confront challenges and accelerates recovery from depletion, thereby reducing ego depletion. Based on the above analysis, this study proposes the following hypotheses:

**H5:** Social mobility beliefs play a negative moderating effect in upward social comparison and ego depletion;

**H5a:** Social mobility beliefs have a negative moderating effect on the relationship between upward social comparison and ego-cognitive depletion;

**H5b:** Social mobility beliefs have a negative moderating effect on the relationship between upward social comparison and ego-emotional depletion;

**H5c:** Social mobility beliefs have a negative moderating effect on the relationship between upward social comparison and ego-behavioral depletion.

As a kind of social cognitive tendency and striving upward expectation, the belief in social mobility can provide individuals with a way of thinking to regulate their attitudes and behaviors and stimulate their motivation (Du et al., 2022). For rural-origin civil servants, achieving results through their own efforts will help to enrich their own resources. The negative cognition of rural-origin civil servants due to social comparison is improved, and upward drive is generated to regulate their goal orientation and pursuit. Studies have shown that a higher belief in social mobility can enhance the subjective wellbeing of individuals, allowing them to experience more positive emotions and weaken negative emotional experiences in deprived environments (Weiss et al., 2022). Moreover, as an expectation of upward social mobility, the belief in social mobility also has motivational effects on individual attitudes and behaviors. To achieve upward mobility to a higher level of society, goal orientation and pursuit can be regulated. For individuals believing that effort can alter fate, work serves not merely as a livelihood but as a critical pathway for upward social mobility. Consequently, even after experiencing upward social comparison and temporary resource depletion, those holding strong beliefs in social mobility exhibit stronger motivation to sustain work engagement and greater reluctance to employ strategies potentially detrimental to career advancement. This directly buffers the impact of ego depletion on work withdrawal behavior. This effect is particularly crucial within the civil service system, characterized by clearly defined promotion pathways.

Hence, the belief in social mobility is introduced as the core moderating variable, grounded in its theoretical capacity to profoundly influence rural-origin civil servants' cognitive appraisal of upward social comparison, emotional responses, resource mobilization, and ultimate behavioral choices. Examining this moderating effect holds significant theoretical value for understanding how individual beliefs shape stress coping patterns and work behaviors against a backdrop of structural disparities. Based on the preceding analysis, the following hypothesis is proposed:

**H6**: Social mobility beliefs have a negative moderating effect on the relationship between upward social comparison and work withdrawal behavior.

**Figure 1:**
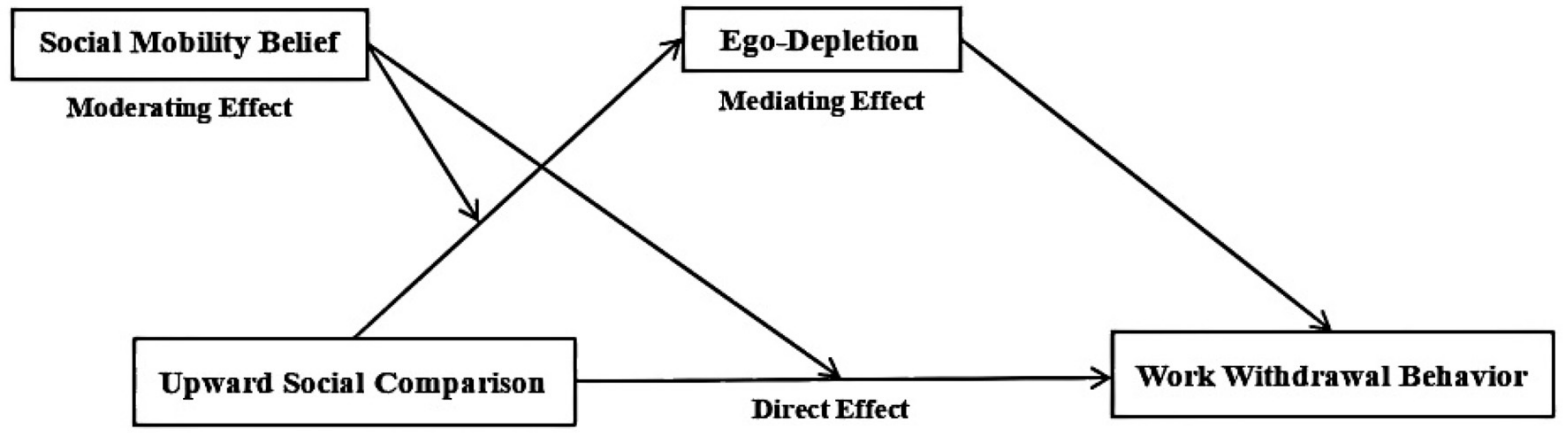
Theoretical analysis model. This figure illustrates the core theoretical model. Within this model, “Upward Social Comparison” serves as the independent variable, “Work Withdrawal Behavior” as the dependent variable, “ego depletion” (encompassing cognitive, emotional, and behavioral dimensions) as the mediator, and “social mobility belief” as the moderator. Solid arrows indicate direct effects or mediating pathways, specifically including: (1) the direct effect of upward social comparison on work withdrawal behavior; (2) the mediating pathways through which upward social comparison affects work withdrawal behavior via the three dimensions of ego depletion; and (3) the direct effects of each ego depletion dimension on work withdrawal behavior. Dashed arrows indicate the negative moderating effect of social mobility belief on the paths from upward social comparison to ego depletion (and its dimensions) and from upward social comparison to work withdrawal behavior.

Drawing upon research related to social comparison theory, ego depletion theory, and the belief in social mobility, and considering the complex identity of rural-origin civil servants, the study proposes the research framework “Upward Social Comparison → Ego Depletion → Work Withdrawal Behavior among Rural-Origin Civil Servants,” as illustrated in Figure 1.

The unique identity background of rural-origin civil servants requires specific consideration when examining the mechanism through which upward social comparison affects their psychology and behavior. This background involves not only institutional differences based on household registration but also persistent challenges in identity adaptation stemming from rural-to-urban migration and occupational transition. Prolonged urban residence may weaken their original identification with a “rural identity”; yet persistent experiences of difference may simultaneously reinforce feelings of marginalization. Consequently, the intensity and manifestation of self-threat, perceived unfairness, and ego depletion triggered by upward social comparison are likely moderated by an individual's current state of identity and the degree of urbanization experienced. This study introduces the concepts of ego depletion and social mobility belief to elucidate how social mobility belief moderates the relationship between upward social comparison and work withdrawal behavior among rural-origin civil servants, mediated by ego depletion.

## 3 Research design

### 3.1 Methods

Data were collected via questionnaires. Procedural controls were implemented during data collection to mitigate potential common method bias. Respondent anonymity was ensured, and participants were informed that there were no correct or incorrect answers. Survey items were arranged modularly, progressing from demographic variables to independent, mediating, dependent, and moderating variables, with items randomized within each module.

Empirical data were collected through questionnaire surveys. SPSS 22.0 was employed for data processing and analysis, including descriptive statistics, correlation analysis, common method bias assessment, and moderated multiple regression analysis to test moderation effects. Amos 21.0 was utilized to construct structural equation models examining direct effects, mediating effects, and overall model fit indices. Amos was selected primarily due to its recognized advantages in latent variable modeling: its graphical interface facilitates the construction and modification of complex theoretical models involving multiple latent variables with associated measurement models and structural paths (Gustafsson and Martenson, 2002); it simultaneously estimates measurement models through confirmatory factor analysis and structural path models while effectively handling measurement error in observed variables, thereby yielding more accurate estimates of relationships between latent variables; it provides comprehensive model fit indices, including χ^2^/df, RMSEA, CFI, and TLI for evaluating the correspondence between theoretical models and empirical data.

The structural equation model incorporated four latent variables—upward social comparison, ego depletion, social mobility belief, and work withdrawal behavior—each measured by multi-item scales.

### 3.2 Survey region

The subjects of this study are rural-origin civil servants who have been employed for 10 years or less in Jinan City, Shandong Province. Jinan is one of the 15 sub-provincial cities in China. As the capital of Shandong Province, its annual recruitment rate for public officials ranks among the top in the province. According to the “Jinan 7th National Population Census Bulletin”, the number of permanent residents in the city with a university (referring to college or above) education level was 2.386 million. Among the permanent residents of the city, there were 3.297 million people separated from household registration, of which 1.462 million were separated from household registration within the municipal district. The ratio of civil servant applicants in Jinan City ranks among the top in Shandong Province every year. Therefore, the research conclusions drawn from the case study of Jinan City have reference value for exploring the work withdrawal behavior of rural-origin civil servants.

The rural-origin civil servants who have been employed for 10 years or less were included in the survey for the following reasons: on the one hand, most of the civil servants have not formed or have just formed a family, have weak social support networks, poor economic foundations, and low professional status, and the lasting influence of social background has not been completely eliminated (Holman and Kokko, 2014). Their work withdrawal behavior is more prominent in the process of work. On the other hand, according to Bourdieu's social reproduction theory, individuals are endowed with different habits in different fields, and these habits will affect people's behavior and thinking patterns (Gardner and Tang, 2014). Civil servants are in the career adaptation and development stage after 10 years of entry or less, while the habits and cognitive residue formed in rural areas have not dissipated completely and will continue to play a role at work.

### 3.3 Data sources

Data Collection: Data were collected using a self-designed “Rural-Origin Civil Servant Behavior Questionnaire.” A combined stratified and random sampling strategy was implemented as follows. Jinan City districts served as the primary sampling units. Districts were stratified by regional economic development level (high, medium, low): Lixia District (high), Shizhong and Licheng Districts (medium), and Changqing District (low). Within each sample district, six institutions were randomly selected: two legal/political institutions (courts, procuratorates, public security bureaus), two government departments (e.g., civil affairs, education bureaus), and two mass organizations (e.g., Communist Youth League committees, Women's Federations). Finally, within each selected institution, 40–60 eligible rural-origin civil servants—defined as individuals holding rural household registration but working in urban Jinan governmental units—were randomly selected.

Interview Procedure: To ensure comprehension and data quality, particularly regarding potentially complex psychological concepts, structured face-to-face interviews were conducted uniformly. Investigators received standardized training covering questionnaire content, core concept definitions, and interview protocols. Each participant underwent a one-on-one interview where investigators read items aloud, providing standardized plain-language explanations of technical terms as needed to ensure accurate understanding. Participants provided responses verbally, and investigators recorded the selected options directly onto paper questionnaires. Interviews were not audio-recorded; only the final quantitative answers were documented. Questionnaires were collected immediately post-interview. Prior to participation, all individuals were informed of the study's purpose, with anonymity and voluntary participation emphasized; verbal informed consent was obtained.

Sample Characteristics and Response Rate: The sample encompassed civil servants of varying genders, educational backgrounds, professional ranks, marital statuses, and work tenures. Representativeness was enhanced as participants originated from various Shandong municipalities, with approximately 13.8% being non-Shandong natives, allowing the sample to reflect broader characteristics of Chinese rural-origin civil servants. Out of 1,400 distributed questionnaires, 1,359 were returned, with 1,137 deemed valid, yielding an 83.6% valid response rate. Raw data underwent entry, cleaning, and strict de-identified processing to create an anonymous dataset for subsequent analysis.

### 3.4 Variable setting and description

To mitigate common method bias and optimize participant experience, the survey questionnaire was modularized and presented sequentially. Demographic information—including gender, age, education level, work tenure, professional title, place of origin, current residence, and the proportion of rural-origin colleagues among others—was collected first to acclimatize participants. Subsequent modules measured upward social comparison, followed by the three dimensions of ego depletion (cognitive, emotional, and behavioral depletion). Work withdrawal behavior was then assessed, with social mobility beliefs measured last. Items within each module were randomly ordered. All items used a five-point Likert scale, with responses ranging from 1 (strongly disagree) to 5 (strongly agree).

#### 3.4.1 Independent variable: upward social comparison

The upward social comparison scale adopted the social comparison tendency scale compiled by (Reer et al. 2019). The scale, originally comprising 14 items measuring upward social comparison in work and life contexts, was adapted for the specific rural civil servant population under investigation. Its internal consistency was excellent, as indicated by a Cronbach's alpha coefficient of 0.956 for the overall sample. All items demonstrated corrected item-total correlations ranging from 0.719 to 0.828, exceeding the 0.5 threshold. Deletion of any item failed to increase the alpha value. Factor analysis suitability was confirmed by a KMO value of 0.975 and a significant Bartlett's test of sphericity, with an approximate chi-square value of 11,798.877, 91 degrees of freedom, and a *p*-value < 0.001. Principal component analysis extracted a single factor with an eigenvalue exceeding 1.0, explaining 63.785% of the cumulative variance, which surpassed the 60% criterion. All factor loadings ranged from 0.736 to 0.838, exceeding 0.5 without cross-loadings, demonstrating a clear scale structure. Confirmatory factor analysis further supported the scale's validity, yielding good model fit indices: χ^2^/df = 4.722, RMSEA = 0.057, GFI = 0.957, AGFI = 0.941, NFI = 0.969, IFI = 0.976, CFI = 0.976, TLI = 0.971. All standardized factor loadings were statistically significant, ranging from 0.736 to 0.828. Composite reliability was 0.956, and the average variance extracted was 0.610, exceeding the 0.5 benchmark, thereby confirming convergent and structural validity.

#### 3.4.2 Mediating variable: ego depletion

State perceived ego depletion was measured in this study using a scale assessing participants' subjectively reported depletion of self-control resources within a work context. Adapted from Vohs et al.'s (2021) design and revised for the civil servant context, this scale measures three dimensions of ego depletion, focusing on perceived internal state changes and diminished capacity following resource expenditure. The ego-cognitive depletion dimension assesses perceived difficulties in cognitive function efficiency, specifically concerning attention concentration, information processing, and decision-making (3 items). The ego-emotional depletion dimension measures perceived emotional resource exhaustion and reduced emotional control capacity resulting from sustained emotion regulation (3 items). The ego-behavioral depletion dimension evaluates perceived impairment in behavioral control capacity, including the inhibition of impulsive actions, resistance to temptation, and adherence to plans or rules (4 items).

The overall scale demonstrated good internal consistency, with a Cronbach's alpha coefficient of 0.865. Dimension-specific reliability was also good: cognitive depletion α = 0.862, emotional depletion α = 0.926, and behavioral depletion α = 0.855. All corrected item-total correlations exceeded 0.5, ranging from 0.735 to 0.760 for cognitive depletion, 0.809 to 0.842 for emotional depletion, and 0.677 to 0.720 for behavioral depletion. Cronbach's alpha for each dimension did not increase following the deletion of any single item, confirming the scale's robust internal consistency.

Factor analysis suitability was indicated by a KMO value of 0.878 and a significant Bartlett's test of sphericity, χ^2^(55) = 7,659.68, p < 0.001. Using principal component analysis with varimax rotation and retaining factors with eigenvalues exceeding 1.0, three factors were extracted. These factors cumulatively explained 76.839% of the variance, exceeding the recommended 60% threshold. All items loaded clearly onto their theoretically proposed dimensions, with factor loadings above 0.5, ranging from 0.820 to 0.832 for cognitive depletion, 0.832 to 0.888 for emotional depletion, and 0.816 to 0.855 for behavioral depletion. No significant cross-loadings were observed, supporting good structural validity.

Excellent model fit indices were obtained: χ^2^/df = 2.739, RMSEA = 0.039, GFI = 0.982, AGFI = 0.971, NFI = 0.985, IFI = 0.991, CFI = 0.991, TLI = 0.987. All indices met established thresholds for good fit. All standardized factor loadings were statistically significant, p < 0.001, ranging from 0.805 to 0.842 for cognitive depletion, with CR = 0.863 and AVE = 0.678; from 0.853 to 0.885 for emotional depletion, with CR = 0.927 and AVE = 0.759; and from 0.741 to 0.809 for behavioral depletion, with CR = 0.857 and AVE = 0.600. The AVE for each dimension exceeded 0.5, indicating good convergent validity.

#### 3.4.3 Moderating variable: social mobility belief

The social mobility belief scale adopted the social mobility belief questionnaire compiled by (Manstead 2018) to examine the subjects' social mobility belief based on their current and future cognition and expectations of their social and economic status. It included 8 items, such as “With the current social situation, I can continuously improve my social status”. The scale demonstrated excellent internal consistency in this sample, with an overall Cronbach's α coefficient of 0.916. All item-total correlations ranged from 0.681 to 0.808, exceeding the 0.5 threshold. Cronbach's α did not increase following the deletion of any individual item. Factor analysis suitability was confirmed by a KMO value of 0.941 and a significant Bartlett's test of sphericity, with an approximate χ^2^ value of 5,225.437, 28 degrees of freedom, and a *p*-value below 0.001. Principal component analysis, using an eigenvalue criterion exceeding 1, extracted a single factor accounting for 63.15% of the total variance, which is above the acceptable threshold. All factor loadings were above 0.5, ranging from 0.710 to 0.857, indicating a clear scale structure. Good model fit was evidenced by the following indices: χ^2^/df = 4.966, RMSEA = 0.059, GFI = 0.978, AGFI = 0.961, NFI = 0.981, IFI = 0.985, CFI = 0.985, and TLI = 0.979. All values met established cutoffs. Furthermore, all standardized factor loadings were significant and ranged from 0.710 to 0.857. Composite reliability was 0.917, and the average variance extracted was 0.581, exceeding the 0.5 benchmark. These results collectively support good convergent and structural validity.

#### 3.4.4 Dependent variable: work withdrawal behavior

The work withdrawal behavior scale compiled by (Aggarwal et al. 2020) and revised by Fan P. et al. (2023) was adopted in this study. The revised scale contained two dimensions: psychological withdrawal and behavioral withdrawal. The questionnaire consisted of 11 questions. Behavioral withdrawal refers to observable actions such as reduced work effort and avoidance of responsibilities, measured by six items. Psychological withdrawal describes an internal state of detachment from work, including inattentiveness and mental disengagement, measured by five items.

The overall Cronbach's alpha coefficient for the work withdrawal scale was 0.904, indicating high internal consistency. The subscale reliabilities were 0.923 for behavioral withdrawal and 0.873 for psychological withdrawal. All corrected item-total correlations exceeded 0.5, ranging from 0.751 to 0.802 for behavioral withdrawal and from 0.657 to 0.743 for psychological withdrawal. Internal consistency was further supported as the alpha coefficient did not increase following the deletion of any single item.

Factor analysis suitability was confirmed by a KMO value of 0.927 and a significant Bartlett's test of sphericity (χ^2^ = 7,614.612, df = 55, *p* < 0.001). Principal component analysis with varimax rotation extracted two factors with eigenvalues exceeding 1.0, collectively accounting for 69.721% of the variance. All items loaded cleanly onto their hypothesized factors, with factor loadings ranging from 0.793 to 0.842 for behavioral withdrawal and from 0.753 to 0.821 for psychological withdrawal, demonstrating clear structure without cross-loadings.

Confirmatory factor analysis indicated good model fit: χ^2^/df = 3.263, RMSEA = 0.045, GFI = 0.979, AGFI = 0.966, NFI = 0.982, IFI = 0.987, CFI = 0.987, TLI = 0.984. All standardized factor loadings were statistically significant (p < 0.001), ranging from 0.786 to 0.843 for behavioral withdrawal (composite reliability = 0.924, average variance extracted = 0.668) and from 0.712 to 0.810 for psychological withdrawal (composite reliability = 0.874, average variance extracted = 0.582). Convergent validity was established as all AVE values exceeded 0.5.

### 3.5 Common method bias test

This study employed a questionnaire survey for data collection. To mitigate potential common method bias, procedural controls were implemented during data gathering. Respondent anonymity was assured, and participants were informed that responses had no right or wrong answers. Questionnaire items were organized using a modular design covering demographic variables, independent variables, mediating variables, dependent variables, and moderating variables. Within each module, items were presented in randomized order rather than being grouped by construct.

This study adopted the most commonly used Harman's single-factor method to test for common method bias. In exploratory factor analysis, the unrotated factor analysis results were examined. The results extracted factors with eigenvalues >1. If the percentage of variance explained by the first factor does not reach 40%, it indicates that common method bias does not have a serious impact on this study. The specific results are shown in Table 1.

**Table 1:** Test of common method bias.

**Component**	**Initial eigenvalues**			**Extraction sums of squared loadings**		
	**Total**	**% of variance**	**Cumulative %**	**Total**	**% of variance**	**Cumulative %**
1	12.675	28.807	28.807	12.675	28.807	28.807
2	6.784	15.419	44.226	6.784	15.419	44.226
3	2.751	6.252	50.477	2.751	6.252	50.477
4	2.668	6.063	56.541	2.668	6.063	56.541
5	2.367	5.379	61.920	2.367	5.379	61.920
6	1.820	4.136	66.056	1.820	4.136	66.056
7	1.218	2.769	68.825	1.218	2.769	68.825
..	..	..	..			
44	0.179	0.408	100			

This study conducted factor analysis on all items and used the unrotated principal component analysis method (i.e., Harman's single-factor test) to evaluate the impact of common method bias. The analysis results showed that the percentage of variance explained by the largest unrotated factor was 28.807%, which is far lower than 40%. This indicates that there is no single factor in the sample data that can explain most of the variance, meaning that there is no serious common method bias in this study.

## 4 Research results

In this study, SPSS 22.0 and AMOS 21.0 were used to conduct an empirical analysis of the data, and a structural equation model was used to examine the effect and its mechanism of upward social comparison on rural-origin civil servants' work withdrawal behavior. Meanwhile, the regression path analysis method of Bootstrap was used to incorporate four variables into the same model to test the significance of the mediating effect and the moderating effect.

### 4.1 Descriptive statistical analysis

Descriptive statistical analysis was conducted on the basic demographic characteristics and work–life perceptions of the valid sample (*N* = 1,137). The results are presented in Table 2.

As shown in Table 2a, the sample comprised slightly more male civil servants (56.1%) than female ones (43.9%). Educational attainment was predominantly at the undergraduate level (44.1%) or college level and below (38.0%), with master's and doctoral degrees representing a smaller proportion (17.9% combined). Most respondents (73.8%) had fewer than 5 years of work experience, indicating a sample primarily composed of younger civil servants with relatively short tenures. The vast majority were married (90.9%). Place of origin was predominantly rural areas within Shandong Province (86.2%), with 13.8% originating from rural areas outside the province. Regarding professional rank, over 70% (75.1%) held positions at the clerical level or below. Current residence was mainly in county-level cities or towns (31.0%) or prefecture-level cities and above (45.8%). A large majority of the sample (84.2%) reported that rural-origin civil servants constituted < 50% of their colleagues.

**Table 2a:** Descriptive statistic results.

**Items**	**Categories**	**Frequency (person)**	**Percentage (%)**
Sex	Male	638	56.1
	Female	499	43.9
Education	Junior college and below	432	38
	Undergraduate	501	44.1
	Postgraduate	171	15
	Doctor	33	2.9
Length of service	< 1 year	210	18.5
	1–3 years	241	21.2
	3–5 years	388	34.1
	5–10	298	26.2
Profession title	Clerk	216	19
	Staff member	638	56.1
	Senior staff member	239	21
	Principal staff member	35	3.1
	Secondary and above	9	0.8
Marital status	Unmarried	103	9.1
	Married	1,034	90.9
Native place	New first-tier city (Qingdao)	205	18
	Second-tier cities (Jinan, Linyi, Weifang, Yantai)	285	25.1
	Third-tier cities (Jining, Zibo, etc.)	307	27
	Fourth-tier cities (Zaozhuang, Rizhao, etc.)	183	16.1
	Outside Shandong Province	157	13.8
Current residence	Villages	138	12.1
	Small town	125	11
	County or county-level city	353	31
	Prefecture-level city and above	521	45.8
Around rural-origin civil servants proportion	Under 10%	137	12
	10%−30%	331	29.1
	30%−50%	489	43
	>50%	180	15.8

Jining, Zibo, Heze, Weihai, Dezhou, Tai'an, and Liaocheng are classified as third-tier cities; Zaozhuang, Rizhao, Binzhou, and Dongying are classified as fourth-tier cities.

As shown in Table 2b, across perceived distributions of job adaptation, life satisfaction, workplace harmony (with non-rural-origin colleagues), future development satisfaction, economic satisfaction, and social satisfaction, over 60% of respondents reported feeling “relatively satisfied” or “very satisfied” with each indicator. However, a significant proportion (~20–40%) indicated “neutral” or “dissatisfied” responses. Negative perceptions were particularly noted concerning job adaptation (20.7% neutral, 12.3% dissatisfied), life satisfaction (24.5% neutral, 14.4% dissatisfied), workplace harmony (26.5% neutral, 12.9% dissatisfied), and future development satisfaction (26.3% neutral, 12.8% dissatisfied).

**Table 2b:** Descriptive statistic results.

**Items frequency categories**	**Very dissatisfied**	**Not satisfied**	**General**	**Quite satisfactory**	**Very satisfied**
Job adaptability	54 (4.7)	86 (7.6)	235 (20.7)	368 (32.4)	394 (34.7)
Life satisfaction	45 (4.0)	118 (10.4)	279 (24.5)	303 (26.6)	392 (34.5)
Work harmony	49 (4.3)	98 (8.6)	301 (26.5)	336 (29.6)	353 (31.0)
Future development satisfaction	39 (3.4)	107 (9.4)	299 (26.3)	371 (32.6)	321 (28.2)
Economic satisfaction	30 (2.6)	100 (8.8)	373 (32.8)	331 (29.1)	303 (26.6)
Social satisfaction	47 (4.1)	109 (9.6)	244 (21.5)	398 (35.0)	339 (29.8)

### 4.2 Correlation analysis of each variable

The means, standard deviations, maximum values, minimum values, and correlation coefficients of the main variables are presented in Table 3. When there was a connection between variables that could not be directly explained by causality, the relationship between them was called correlation. This study first analyzed the relationship between variables in this study through Pearson correlation. For the six latent variables included in this study (except for the upward social comparison, which had no significant correlation with ego-behavioral depletion, with *p* > 0.05), the *p*-values corresponding to correlation coefficients of all other latent variables were < 0.01, indicating significant statistical significance. These results indicated that upward social comparison was correlated with work withdrawal behavior, ego depletion, and social mobility belief. These correlations were generally consistent with the expectations of this study and provided a preliminary basis for subsequent hypotheses.

**Table 3:** Descriptive statistics and correlation analysis of main variables.

**Variable**	**M**	**SD**	**1**	**2**	**3**	**4**	**5**	**6**
1. Upward social comparison	3.681	0.825	1					
2. Ego-cognitive depletion	3.367	0.865	0.195^**^	1				
3. Ego-emotional depletion	3.002	0.971	0.223^**^	0.559^**^	1			
4. Ego-behavioral depletion	3.495	0.886	0.032	0.209^**^	0.242^**^	1		
5. Work withdrawal behavior	3.814	0.732	0.305^**^	0.416^**^	0.430^**^	0.249^**^	1	
6. Social mobility belief	3.698	0.807	0.213^**^	0.386^**^	0.385^**^	0.271^**^	0.505^**^	1

^*^P < 0.05, ^**^P < 0.01, ^***^P < 0.001.

### 4.3 Hypothesis testing based on structural equation model

Based on the correlation analysis of variables, Amos 21.0 was applied to test the hypothesis relationship between variables by constructing a structural equation model.

#### 4.3.1 Structural equation model test results

**Figure 2:**
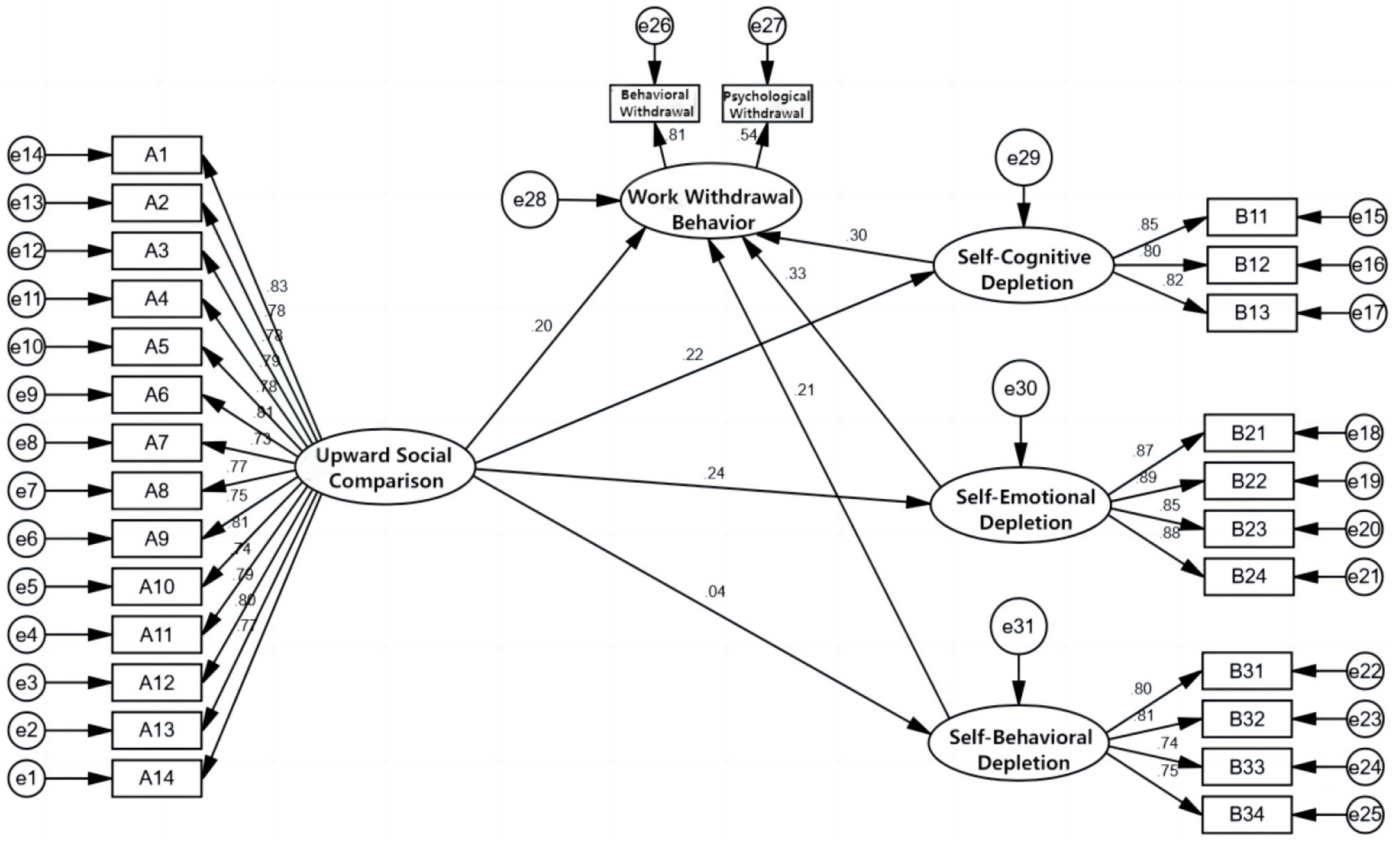
Structural equation model diagram.

To verify the direct effect of upward social comparison on work withdrawal behavior and the mediating effect of ego depletion of rural-origin civil servants, a structural equation model was constructed. Upward social comparison was the independent variable, ego depletion was the mediating variable, and work withdrawal behavior was the dependent variable (Figure 2). When judging whether the structural equation model was valid, it was mainly measured by the assessment of some fitting indices. χ^2^/df was generally required to be < 5; GFI was the goodness-of-fit index, AGFI was the adjusted goodness-of-fit index, NFI was the normed fit index, TLI was the Tucker–Lewis index, and CFI was the comparative fit index. These values were generally required to be >0.9, indicating that the model adaptability was good; a value >0.8 indicated that the model was acceptable. RMSEA should be < 0.08, indicating good adaptation ability and a good model fit.

As can be seen from Table 4, the χ^2^/df value was 3.916, < 5. The root mean square error of approximation (RMSEA) was 0.051, less than the standard level of 0.08, indicating a good fit. GFI = 0.927, AGFI = 0.913, NFI = 0.940, IFI = 0.954, CFI = 0.954, TLI = 0.949. The model fitting indices were all within the recommended value range, indicating that the structural equation model established in this study was effective and well matched with the recovered data and could be further explained.

**Table 4:** Structural equation model fitting index.

**Fit index**	**Criteria**	**Value**
χ^2^/df	< 5	3.916
GFI	>0.8	0.927
AGFI	>0.8	0.913
NFI	>0.8	0.940
IFI	>0.8	0.954
CFI	>0.9	0.954
NNFI (TLI)	>0.9	0.949
RMSEA	< 0.08	0.051

#### 4.3.2 Mediating effect hypothesis test

In this study, AMOS 21.0 software was used to analyze the path of the structural equation model, and the path coefficient and C.R. of the structural equation model were obtained. The path coefficient reflected the direction and magnitude of the relationship between variables. The C.R. (Critical Ratio) can determine whether the regression coefficient is significant or not. It is generally believed that a C.R. value ≥1.96 indicates a significant difference at a significance level of 0.05 (Kim et al., 2001).

According to the path analysis results in Table 5, it can be seen that the standardized path coefficient of upward social comparison on work withdrawal behavior was 0.202 (C.R. value = 5.874, *p* < 0.001), indicating that upward social comparison had a significant positive effect on work withdrawal behavior. Thus, hypothesis H1 was valid.

**Table 5:** Path analysis result.

**Path**			**γ**	**S.E**.	**C.R**.	***P***
Work withdrawal behavior	< –	Upward social comparison	0.202	0.028	5.874	^***^
Ego-cognitive depletion	< –	Upward social comparison	0.219	0.033	6.709	^***^
Ego-emotional depletion	< –	Upward social comparison	0.239	0.038	7.609	^***^
Ego-behavioral depletion	< –	Upward social comparison	0.043	0.031	1.327	0.185
Work withdrawal behavior	< –	Ego-cognitive depletion	0.303	0.028	8.593	^***^
Work withdrawal behavior	< –	Ego-emotional depletion	0.333	0.023	9.721	^***^
Work withdrawal behavior	< –	Ego-behavioral depletion	0.211	0.029	6.199	^***^

^*^P < 0.05, ^**^P < 0.01, ^***^P < 0.001.

The standardized path coefficient of ego-cognitive depletion on work withdrawal behavior was 0.303 (C.R. value = 8.593, *p* < 0.001), indicating that ego-cognitive depletion had a significant positive effect on work withdrawal behavior. Hypothesis H3a was assumed to be valid. The standardized path coefficient of ego-emotional depletion on work withdrawal behavior was 0.333 (C.R. value = 9.721, *p* < 0.001), indicating that ego-emotional depletion had a significant positive effect on work withdrawal behavior. Hypothesis H3b was valid. The standardized path coefficient of ego-behavioral depletion on work withdrawal behavior was 0.211 (C.R. value = 6.199, *p* < 0.001), indicating that ego-behavioral depletion had a significant positive effect on work withdrawal behavior; thus, hypothesis H3c was valid.

The standardized path coefficient of upward social comparison on ego-cognitive depletion was 0.219 (C.R. value = 6.709, *p* < 0.001), indicating that upward social comparison had a significant positive effect on cognitive loss; thus, hypothesis H2a was valid. The standardized path coefficient of upward social comparison on ego-emotional depletion was 0.239 (C.R. value = 7.609, *p* < 0.001), indicating that upward social comparison had a significant positive effect on ego-emotional depletion; thus, hypothesis H2b was valid. The standardized path coefficient of upward social comparison on ego-behavioral depletion was 0.043 (C.R. value = 1.327, *p* > 0.05), indicating that upward social comparison had no significant effect on ego-behavioral depletion; therefore, hypothesis H2c was not valid.

The bootstrap method in AMOS was used to test the mediating effect, with 5,000 samples repeated to calculate the 95% confidence interval. From the results in Table 6, the total effect value of the mediation path [upward social comparison-work withdrawal behavior] was 0.357, and the upper and lower intervals of the 95% confidence interval were positive, excluding 0. The *P*-value was less than the significance level of 0.05, indicating that the total effect existed. The direct effect value was 0.202, and the upper and lower intervals of the 95% confidence interval were both positive, excluding 0. The *P*-value was less than the significance level of 0.05, indicating the existence of a direct effect, accounting for 56.58% of the total effect. Therefore, ego depletion played a mediating role in upward social comparison and work withdrawal behavior, confirming that hypothesis H4 was valid. The effect size of the first indirect pathway (upward social comparison → ego-cognitive depletion → work withdrawal behavior) was 0.066, and the upper and lower intervals of the 95% confidence interval were positive, excluding 0. The *P*-value was less than the significance level of 0.05, indicating the existence of indirect effect 1, accounting for 18.49% of the total effect. Therefore, hypothesis H4a was proved to be true. The effect value of indirect effect 2 [upward social comparison—ego-emotional depletion—work withdrawal behavior] was 0.080, and the upper and lower intervals of the 95% confidence interval were positive, excluding 0. The *P*-value was less than the significance level of 0.05, indicating the existence of indirect effect 2, accounting for 22.41% of the total effect. Therefore, hypothesis H4b was proved to be true. The effect value of indirect effect 3 [upward social comparison—self-behavior depletion—work withdrawal behavior] was 0.009, and the upper and lower intervals of the 95% confidence interval included both positive and negative values, including 0. The *P*-value was greater than the significance level of 0.05, indicating that indirect effect 3 did not exist, accounting for 2.52% of the total effect. Therefore, it was concluded that hypothesis H4c was not valid.

**Table 6:** Bootstrap for mediating effect test.

**Effect pathway**	**Effect type**	**Effect value**	**SE**	**95% CI**		***P***	**Proportion**
				**Lower**	**Upper**		
Upward social comparison-work withdrawal behavior	Total effect	0.357	0.053	0.25	0.457	0.000	-
	Direct effect	0.202	0.045	0.115	0.291	0.000	56.58%
Upward social comparison-ego-cognitive depletion-work withdrawal behavior	Indirect effect	0.066	0.014	0.041	0.099	0.000	18.49%
Upward social comparison-ego-emotional depletion-work withdrawal behavior	Indirect effect	0.080	0.012	0.057	0.106	0.000	22.41%
Upward social comparison-ego-behavioral depletion-work withdrawal behavior	Indirect effect	0.009	0.007	−0.004	0.023	0.17	2.52%

The bootstrap method in AMOS was used to test the mediating effect, with 5,000 samples repeated to calculate the 95% confidence

interval. From the results in Table 6, the total effect value of the mediation path [upward social comparison-work withdrawal behavior] was 0.357, and the upper and lower intervals of the 95% confidence interval were positive, excluding 0. The *P*-value was less than the significance level of 0.05, indicating that the total effect existed. The direct effect value was 0.202, and the upper and lower intervals of the 95% confidence interval were both positive, excluding 0. The *P*-value was less than the significance level of 0.05, indicating the existence of a direct effect, accounting for 56.58% of the total effect. Therefore, ego depletion played a mediating role in upward social comparison and work withdrawal behavior, confirming that hypothesis H4 was valid. The effect size of the first indirect pathway (upward social comparison → ego-cognitive depletion → work withdrawal behavior) was 0.066, and the upper and lower intervals of the 95% confidence interval were positive, excluding 0. The *P*-value was less than the significance level of 0.05, indicating the existence of indirect effect 1, accounting for 18.49% of the total effect. Therefore, hypothesis H4a was proved to be true. The effect value of indirect effect 2 [upward social comparison—ego-emotional depletion—work withdrawal behavior] was 0.080, and the upper and lower intervals of the 95% confidence interval were positive, excluding 0. The *P*-value was less than the significance level of 0.05, indicating the existence of indirect effect 2, accounting for 22.41% of the total effect. Therefore, hypothesis H4b was proved to be true. The effect value of indirect effect 3 [upward social comparison—self-behavior depletion—work withdrawal behavior] was 0.009, and the upper and lower intervals of the 95% confidence interval included both positive and negative values, including 0. The *P*-value was greater than the significance level of 0.05, indicating that indirect effect 3 did not exist, accounting for 2.52% of the total effect. Therefore, it was concluded that hypothesis H4c was not valid. The bootstrap method in AMOS was used to test the mediating effect, with 5,000 samples repeated to calculate the 95% confidence interval. From the results in Table 6, the total effect value of the mediation path [upward social comparison-work withdrawal behavior] was 0.357, and the upper and lower intervals of the 95% confidence interval were positive, excluding 0. The *P*-value was less than the significance level of 0.05, indicating that the total effect existed. The direct effect value was 0.202, and the upper and lower intervals of the 95% confidence interval were both positive, excluding 0. The *P*-value was less than the significance level of 0.05, indicating the existence of a direct effect, accounting for 56.58% of the total effect. Therefore, ego depletion played a mediating role in upward social comparison and work withdrawal behavior, confirming that hypothesis H4 was valid. The effect size of the first indirect pathway (upward social comparison → ego-cognitive depletion → work withdrawal behavior) was 0.066, and the upper and lower intervals of the 95% confidence interval were positive, excluding 0. The P-value was less than the significance level of 0.05, indicating the existence of indirect effect 1, accounting for 18.49% of the total effect. Therefore, hypothesis H4a was proved to be true. The effect value of indirect effect 2 [upward social comparison - ego-emotional depletion - work withdrawal behavior] was 0.080, and the upper and lower intervals of the 95% confidence interval were positive, excluding 0. The P-value was less than the significance level of 0.05, indicating the existence of indirect effect 2, accounting for 22.41% of the total effect. Therefore, hypothesis H4b was proved to be true. The effect value of indirect effect 3 [upward social comparison—self-behavior depletion—work withdrawal behavior] was 0.009, and the upper and lower intervals of the 95% confidence interval included both positive and negative values, including 0. The *P*-value was greater than the significance level of 0.05, indicating that indirect effect 3 did not exist, accounting for 2.52% of the total effect. Therefore, it was concluded that hypothesis H4c was not valid.

#### 4.3.3 Moderating effect hypothesis test

(1) The moderating effect of social mobility beliefs on upward social comparison and work withdrawal behavior.

This study used Model 1 in the built-in Process plugin of SPSS 25 software to test the moderating effect. The results showed that in the model with upward social comparison as the independent variable, social mobility belief as the moderating variable, and work withdrawal behavior as the dependent variable, the impact coefficient of the interaction term between upward social comparison and social mobility belief on work withdrawal behavior was −0.139, with a significance level of < 0.01, and the 95% confidence interval did not include 0 (Table 7). It passed the significance test, indicating that social mobility belief has a significant moderating effect on the relationship between upward social comparison and work withdrawal behavior. The coefficient is negative, indicating a negative moderating effect, and Hypothesis H6 is supported.

**Table 7:** The moderating effect of social mobility belief on upward social comparison and work withdrawal behavior.

**Variable**	**Effect**	**SE**	**t**	***p***	**95% confidence interval**	
					**LLCI**	**ULCI**
Upward social comparison	0.152	0.023	6.604	0.000	0.107	0.197
Social mobility belief	0.408	0.023	17.826	0.000	0.363	0.453
Upward social comparison ^*^ social mobility belief	−0.139	0.024	−5.752	0.000	−0.186	−0.091
Low social mobility belief (M-1SD)	0.264	0.026	10.007	0.000	0.212	0.315
Middle social mobility belief (Mean)	0.152	0.023	6.604	0.000	0.107	0.197
High social mobility belief (M+1SD)	0.040	0.033	1.190	0.234	−0.026	0.105

**Figure 3:**
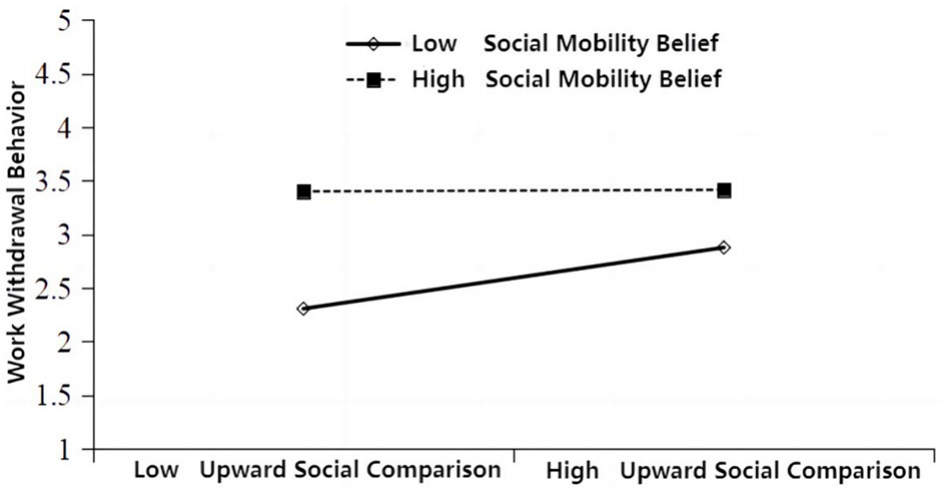
Moderating effect diagram.

Furthermore, according to the effect results under different levels of the moderating variable: under the condition of low social mobility belief (M-1SD), the effect value of upward social comparison on work withdrawal behavior was 0.264, and the 95% confidence interval did not include 0, indicating that the moderating effect was significantly established; under the condition of moderate social mobility belief (Mean), the effect value of upward social comparison on work withdrawal behavior was 0.152, and the 95% confidence interval did not include 0, indicating that the moderating effect was significantly established; under the condition of high social mobility belief (M+1SD), the effect value of upward social comparison on work withdrawal behavior was 0.040, and the 95% confidence interval included 0 (with one positive and one negative value), indicating that the direct effect was not established when the moderating variable was at a high level. Therefore, it is fully proven that as the value of the moderating variable increases, the positive impact of upward social comparison on work withdrawal behavior becomes weaker. These results indicate a significant negative moderating effect of social mobility belief on the relationship between upward social comparison and work withdrawal behavior, supporting H6. The moderating effect is illustrated in Figure 3.

As can be seen from the simple slope chart (Figure 2), when the social mobility belief was low (solid line), the upward social comparison and work withdrawal behavior were positively correlated. When the value of the moderator variable was high (dashed line), the slope of the action direction decreased. This showed that the independent variable had a negative moderating effect on the dependent variable in the case of high adjustment. Specifically, social mobility belief had a significant negative moderating effect on the relationship between upward social comparison and work withdrawal behavior, weakening the positive relationship between upward social comparison and work withdrawal behavior.

(2) The moderating effect of social mobility belief on upward social comparison and ego-cognitive depletion.

This study used Model 1 in the built-in Process plugin of SPSS 25 software to test the moderating effect. The results showed that in the model with upward social comparison as the independent variable, social mobility belief as the moderating variable, and cognitive exhaustion as the dependent variable, the impact coefficient of the interaction term between upward social comparison and social mobility belief on cognitive exhaustion was −0.084, with a significance level < 0.01, and the 95% confidence interval did not include 0 (Table 8). It passed the significance test, indicating that social mobility belief has a significant moderating effect on the relationship between upward social comparison and cognitive exhaustion. The coefficient is negative, indicating a negative moderating effect, and hypothesis H5a is supported.

**Table 8:** The moderating effect of social mobility belief on upward social comparison and ego-cognitive depletion.

**Variable**	**Effect**	**SE**	**t**	**p**	**95% Confidence Interval**	
					**LLCI**	**ULCI**
Upward social comparison	0.104	0.030	3.481	0.001	0.045	0.163
Social mobility belief	0.381	0.030	12.768	0.000	0.322	0.439
Upward social comparison ^*^ social mobility belief	−0.084	0.031	−2.668	0.008	−0.145	−0.022
Low social mobility belief (M-1SD)	0.172	0.034	5.006	0.000	0.104	0.239
Middle social mobility belief (Mean)	0.104	0.030	3.481	0.001	0.045	0.163
High social mobility belief (M+1SD)	0.037	0.044	0.839	0.402	−0.049	0.122

Furthermore, according to the effect results under different levels of the moderating variable: under the condition of low social mobility belief (M-1SD), the effect value of upward social comparison on cognitive exhaustion was 0.172, and the 95% confidence interval did not include 0, indicating that the moderating effect was significantly established; under the condition of moderate social mobility belief (Mean), the effect value of upward social comparison on cognitive exhaustion was 0.104, and the 95% confidence interval did not include 0, indicating that the moderating effect was significantly established; under the condition of high social mobility belief (M+1SD), the effect value of upward social comparison on cognitive exhaustion was 0.037, and the 95% confidence interval included 0 (with one positive and one negative value), indicating that the direct effect was not established when the moderating variable was at a high level. Therefore, it is fully proven that as the value of the moderating variable increases, the positive impact of upward social comparison on cognitive exhaustion becomes weaker. Thus, it was demonstrated that the moderating variable social mobility belief had a significant negative moderating effect on the influence of upward social comparison on ego-cognitive depletion, validating hypothesis H5a.

**Figure 4:**
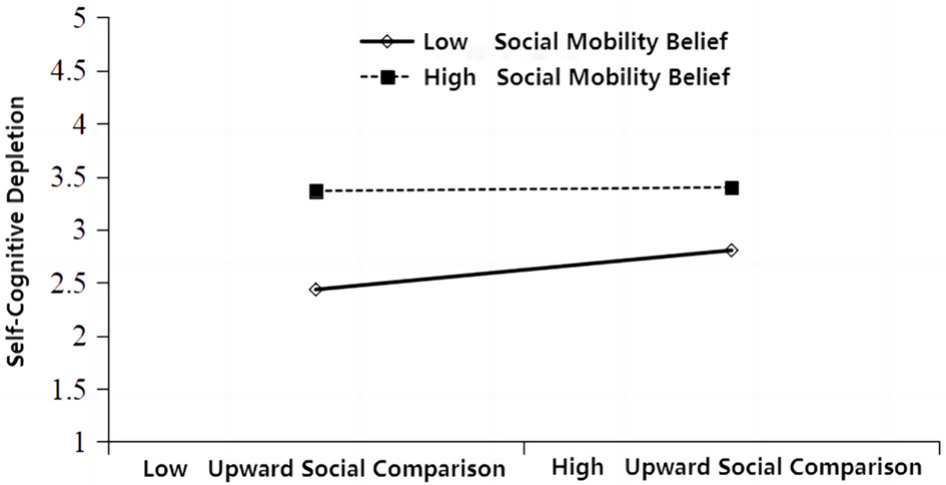
Moderating effect diagram.

As can be seen from the simple slope chart (Figure 4), when the social mobility belief was low (solid line), there was a positive relationship between upward social comparison and ego-cognitive depletion. When the value of the moderator variable was high (dashed line), the slope of the action direction decreased. This showed that the independent variable had a negative moderating effect on the dependent variable in the case of high regulation. Thus, social mobility belief had a significant negative moderating effect on the relationship between upward social comparison and ego-cognitive depletion, weakening the positive relationship between upward social comparison and ego-cognitive depletion.

(3) The moderating effect of social mobility belief on upward social comparison and ego-emotional depletion.

This study used Model 1 in the built-in Process plugin of SPSS 25 software to test the moderating effect. The results showed that in the model with upward social comparison as the independent variable, social mobility belief as the moderating variable, and emotional exhaustion as the dependent variable, the impact coefficient of the interaction term between upward social comparison and social mobility belief on emotional exhaustion was −0.015, with a significance *p*-value of 0.666 (>0.05), and the 95% confidence interval included 0 (Table 9). It did not pass the significance test, indicating that the moderating effect of social mobility belief on the relationship between upward social comparison and emotional exhaustion did not reach a significant level. Therefore, it was concluded that the moderating variable social mobility belief had no significant moderating effect on the influence of upward social comparison on ego-emotional depletion, so the hypothesis H5b was not supported.

**Table 9:** The moderating effect of social mobility belief on upward social comparison and ego-emotional depletion.

**Variable**	**Effect**	**SE**	**t**	***p***	**95% confidence interval**	
					**LLCI**	**ULCI**
Upward social comparison	0.170	0.034	5.055	0.000	0.104	0.235
Social mobility belief	0.424	0.033	12.690	0.000	0.359	0.490
Upward social comparison ^*^ social mobility belief	−0.015	0.035	−0.432	0.666	−0.084	0.054

(4) The moderating effect of social mobility belief on upward social comparison and ego-behavioral depletion.

This study used Model 1 in the built-in Process plugin of SPSS 25 software to test the moderating effect. The results showed that in the model with upward social comparison as the independent variable, social mobility belief as the moderating variable, and behavioral exhaustion as the dependent variable, the impact coefficient of the interaction term between upward social comparison and social mobility belief on behavioral exhaustion was −0.071, with a significance level < 0.05, and the 95% confidence interval did not include 0 (Table 10). It passed the significance test, indicating that social mobility belief has a significant moderating effect on the relationship between upward social comparison and behavioral exhaustion. The coefficient is negative, indicating a negative moderating effect, and Hypothesis H5c is supported.

**Table 10:** The moderating effect of social mobility belief on upward social comparison and ego-behavioral depletion.

**Variable**	**Effect**	**SE**	**t**	***p***	**95% Confidence Interval**	
					**LLCI**	**ULCI**
Upward social comparison	−0.045	0.032	−1.399	0.162	−0.108	0.018
Social mobility belief	0.298	0.032	9.282	0.000	0.235	0.361
Upward social comparison ^*^ social mobility belief	−0.071	0.034	−2.090	0.037	−0.137	−0.004
Low social mobility belief (M-1SD)	0.012	0.037	0.323	0.747	−0.061	0.085
Middle social mobility belief (Mean)	−0.045	0.032	−1.399	0.162	−0.108	0.018
High social mobility belief (M+1SD)	−0.102	0.047	−2.177	0.030	−0.194	−0.010

Furthermore, according to the effect results under different levels of the moderating variable: under the condition of low social mobility belief (M-1SD), the effect value of upward social comparison on behavioral exhaustion was 0.012, and the 95% confidence interval included 0 (with one positive and one negative value), indicating that the moderating effect did not reach a significant level; under the condition of moderate social mobility belief (Mean), the effect value of upward social comparison on behavioral exhaustion was −0.045, and the 95% confidence interval included 0 (with one positive and one negative value), indicating that the moderating effect did not reach a significant level; under the condition of high social mobility belief (M+1SD), the effect value of upward social comparison on behavioral exhaustion was −0.102, and the 95% confidence interval did not include 0, indicating that the moderating effect was significantly established. Therefore, it is fully proven that as the value of the moderating variable increases, the positive impact of upward social comparison on behavioral exhaustion becomes weaker. Therefore, it was proved that the moderating variable social mobility belief had a significant negative moderating effect on the influence of upward social comparison on self-behavior depletion, so the hypothesis H5c was valid.

**Figure 5:**
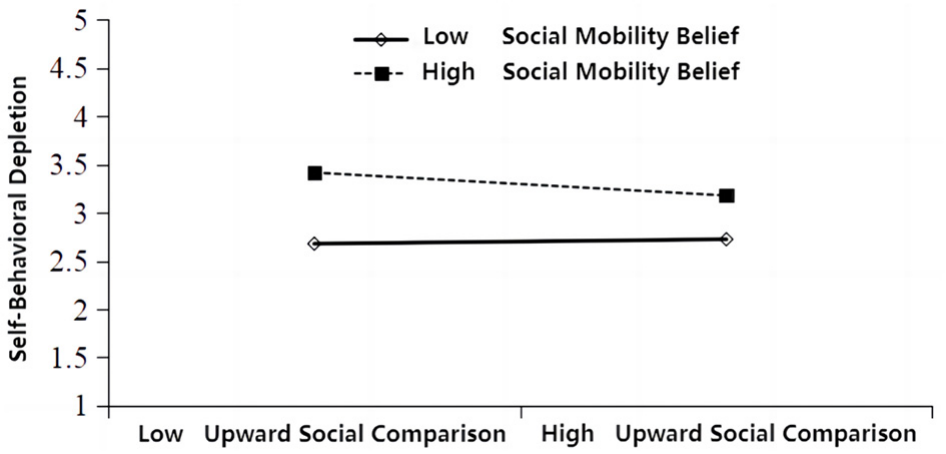
Moderating effect diagram.

As can be seen from the simple slope chart (Figure 5), when social mobility belief was low (solid line), there was a positive relationship between upward social comparison and ego-behavioral depletion. When the value of the moderator variable was high (dashed line), the direction of action changed. It showed that the independent variable had a negative moderating effect on the dependent variable in the case of high regulation. That is, social mobility belief had a significant negative moderating effect on the influence of upward social comparison on ego-behavioral depletion, and social mobility belief weakened the positive relationship between upward social comparison and ego-behavioral depletion.

## 5 Conclusions and recommendations

### 5.1 Discussion

This article focused on the issue of work withdrawal behavior of rural-origin civil servants and conducted a theoretical framework of “upward social comparison—ego depletion—work withdrawal behavior” moderated by social mobility beliefs. A structural equation model was used to analyze the effects of upward social comparison on work withdrawal behavior. On this basis, the Bootstrap method was used to verify the mediating effect of various dimensions of ego depletion (ego-cognitive depletion, ego-emotional depletion, and ego-behavioral depletion), as well as the moderating effect of social mobility belief. The main conclusions were as follows.

#### 5.1.1 Direct effect

The research results show that upward social comparison has a significant impact on work withdrawal behavior, which is consistent with the research findings of (Zhang et al. 2025). Rural-origin civil servants perceive that the feedback received by others is higher than their own during the comparison process, and they subjectively believe that their own effort is greater than the return. They feel that others have gained more trust, attention, and respect, resulting in a negative psychological experience of being treated unfairly by the organization. Especially when compared with non-rural-origin civil servants, the double comparison between identity and achievement makes rural-origin civil servants more sensitive to feelings of inferiority, resulting in a sense of work threat and insecurity and exacerbating the sense of organizational injustice. To balance their own investment and return, eliminate or alleviate such unfair experiences, and vent their dissatisfaction with work, they are prone to different degrees of work withdrawal behavior (Boissicat et al., 2022).

#### 5.1.2 Mediating effect

This validates the research conclusions of Chen, Ming, and others. First, Chen L. et al. (2024) findings revealed that upward social comparisons can engender feelings of workplace envy and ego depletion. Second, (Ming et al. 2020) holds that ego depletion can induce deviant behaviors among subordinates.

Ego depletion played a positive mediating effect between upward social comparison and work withdrawal behavior, which was embodied in ego-cognitive depletion and ego-emotional depletion. The order of effects was: ego-emotional depletion > ego-cognitive depletion. This is because upward social comparison reduces individuals' psychological capital and increases anxiety, resulting in a discrepancy between personal expectations and actual circumstances. At work, when reality is inconsistent with expectations or the psychological contract breaks down, rural-origin civil servants need to use limited self-resources to deal with negative emotions, resulting in ego depletion. Ego depletion, on the other hand, can lead to impulsive and risky behaviors. As a risky behavior, work withdrawal is a “low-cost” and “low-risk” behavior for rural-origin civil servants, providing instant satisfaction in the moment. However, public officials, driven by their own interests such as abiding by discipline and law and conforming to social norms, must invest more control resources into emotional regulation. Therefore, the effect of ego-emotional depletion was greater than that of ego-cognitive depletion. The reason the mediating effect of ego-behavioral depletion was not significant may be due to the special nature of the status of civil servants. Under the professional standards and moral requirements of civil servants, they need to have strong public service motivation and pro-social preferences. Thus, civil servants must embody the spirit of public service and a democratic spirit to ensure the wellbeing of society and the people. Therefore, despite experiencing cognitive and emotional ego depletion and consuming more self-control resources, rural-origin civil servants' strong professional and personal ethics constrain the emergence of impulsive and risky behaviors.

#### 5.1.3 Moderating effect

Existing research findings have not directly proven whether social mobility belief moderates the relationships between upward social comparison, work withdrawal behavior, and ego depletion. However, this study is consistent with some existing research results. First, the research by (Lu and Kandilov 2022) has shown that social mobility belief has a positive impact on life satisfaction. Second, (Schuck and Shore 2019) found that social mobility influences the ways in which young adults view the so-called moral dimension of work and welfare. These studies have demonstrated that social mobility belief can effectively alleviate negative emotions in work and life, which supports the findings of this study.

(1) Social mobility belief negatively moderated the relationship between upward social comparison and ego-cognitive depletion and ego-behavioral depletion. The possible reason is that social mobility belief is a subjective judgment and expectation of individuals on whether upward social mobility can be achieved through their own efforts (Shane and Heckhausen, 2017). As a kind of social cognitive tendency, the expectation of the individual to strive upward can regulate their attitude and behavior by providing a way of thinking. To achieve upward mobility to a higher social level, individuals can regulate their goal orientation and pursuits. The stratification of society by the different social resources possessed by individuals can stimulate the expectation of change (social mobility), that is, a change in social status (Han et al., 2023). For rural-origin civil servants, achieving good results through work input will help to enrich their own resources, provide opportunities for class mobility, improve social status, and enhance their achievement goal orientation. Therefore, rural-origin civil servants with high social mobility belief will transform the differences generated by upward social comparison into the motivation for upward mobility, improve happiness, and change their attitudes and behaviors.

(2) Social mobility beliefs do not have a moderating effect between upward social comparison and ego-emotional depletion. The reason is that the Chinese habitually regard the reference group that has competition in daily life and is superior to their own as the object of comparison, which leads to the threat effect of social comparison (Berendt et al., 2023). The threat effect of upward social comparison refers to the distress, frustration, disappointment, or potential danger that individuals experience when actively searching for information (Fleischmann et al., 2021). Although social mobility beliefs will regulate attitudes and actions, the differences in identity, social background, behavioral patterns, and lifestyles between rural-origin civil servants and non-rural civil servants exist objectively. It still needs a certain process to realize the real transformation of rural-origin civil servants. The appearance of differences or the phenomenon of implicit discrimination in the workplace will cause emotional fluctuations for rural-origin civil servants.

(3) Social mobility belief negatively moderated the relationship between upward social comparison and work withdrawal behavior. The possible reason is that social mobility belief reflects an individual's subjective perception of the degree of macro social justice and fairness, as well as the expectation of upward mobility. Rural-origin civil servants with high social mobility beliefs believe that society is just, democratic, and offers equal opportunities. The more they believe that their social status can be improved through personal efforts, the more willing they are to put energy and time into work. The identity and social status of rural-origin civil servants cannot be changed, but rural-origin civil servants with a higher social mobility belief often have greater expectations for their social status improvement. Upward social comparison makes them not only not produce jealousy, frustration, and other emotions, but also motivates them to work harder. They believe that through acquired efforts, they can change their social class.

#### 5.1.4 Non-significant paths

(1) Absence of Direct Effect on Behavioral Depletion. No significant direct effect of upward social comparison on behavioral depletion was observed, contrary to expectations. Potential explanations include: First, the strong normative constraints associated with the “civil servant” identity may impose stricter institutional regulations, professional ethics, and organizational discipline on overt behaviors than on cognitive or emotional responses. Negative cognitions and emotions resulting from comparison may be suppressed at the behavioral level by a strong professional identity, job security concerns, and fear of disciplinary consequences. The inherent rule-awareness and risk aversion within the civil service system may constitute a “protective barrier” inhibiting impulsive actions. Second, the resilience and traditionalism linked to rural backgrounds may foster greater endurance and pragmatism. Faced with comparison-induced stress, rural-origin civil servants may adopt strategies emphasizing compliance and self-preservation, channeling efforts into diligent work rather than detectable, career-jeopardizing impulsive behaviors. This internalized behavioral regulation reduces the likelihood of upward social comparison directly triggering a behavioral loss of control. Third, the measurement sensitivity of behavioral depletion should be considered. The behavioral depletion scale used primarily measured general impulsive tendencies. Within civil servant populations, the occurrence of such extreme behaviors may inherently be low, or respondents may underreport due to social desirability bias, limiting this dimension's sensitivity to upward social comparison. Future research could explore more context-specific manifestations like “micro-withdrawal” or quasi-compliant behaviors as indicators of behavioral depletion.

(2) Non-Significant Mediation via Behavioral Depletion. The indirect effect of upward social comparison on work withdrawal behavior through behavioral depletion was non-significant. This was expected given the non-significant direct effect on behavioral depletion itself. Results indicate that within this cohort, despite substantial consumption of cognitive and emotional resources, robust professional norms and pragmatic tendencies may effectively suppress overt impulsive actions. However, these factors appear insufficient to prevent cognitive biases, negative emotions, and the subsequent covert work withdrawal behaviors they trigger. This underscores the importance of differentiating depletion dimensions when analyzing complex organizational behaviors. It further supports the preceding analysis regarding the unique nature of behavioral depletion among civil servants: the psychological resource depletion triggered by upward social comparison likely operates primarily at cognitive and emotional levels, directly or indirectly (via cognitive/emotional depletion) influencing work withdrawal behavior, rather than first inducing generalized behavioral loss of control preceding work withdrawal.

(3) Non-Moderating Effect of Social Mobility Belief. Social mobility belief did not buffer the negative impact of upward social comparison on emotional depletion. This suggests two points: First, the immediacy and primacy of emotional responses may render them less amenable to modulation by subjective beliefs than cognitive appraisals or subsequent behavioral tendencies. Negative emotions arising from the direct perception of significant disparity with advantaged others may be difficult to rapidly alleviate by beliefs about future mobility, particularly when the disparity relates to immutable background factors. Second, the cumulative nature and context sensitivity of emotional depletion should be noted. Emotional depletion reflects the draining of psychological resources by sustained negative affect. Social mobility belief, as a relatively stable cognitive disposition, may offer limited efficacy in mitigating the immediate emotional impact of discrete comparison events. Alternatively, its effect may be overwhelmed by persistent workplace stressors, subtle interpersonal “micro-inequities,” or identity conflicts. The complex interplay of competition and cooperation within civil service organizations may facilitate the repeated activation and accumulation of comparison-induced negative emotions, exceeding the regulatory capacity of social mobility beliefs.

### 5.2 Recommendations

Interventions to mitigate work withdrawal behavior among rural-origin civil servants are recommended in the following aspects.

(1) Develop targeted and systematic psychological support and value guidance systems.

Given the identified significant links between upward social comparison and perceived self-cognitive/emotional depletion and work withdrawal behavior, along with the mediating role of perceived depletion, collaboration with professional psychological institutions is required. Regular mental health assessments and risk evaluations must be conducted for all rural-origin civil servants, with dynamic psychological profiles established. Based on assessment outcomes, tiered interventions should be implemented: For widespread issues like identity conflicts, mandatory online learning modules and workshops integrating “civil servant role norms” and “urban–rural integration values” must be developed, utilizing real cases to avoid didactic content. For high-risk individuals or specific groups (e.g., new recruits, promotion-delayed staff), confidential one-on-one counseling pathways facilitated by culturally competent professionals should be established. Concurrently, primary leadership must consistently advocate for a “merit-based” and “equality-respecting” organizational culture in key meetings and daily operations. Discriminatory remarks or behaviors based on origin must be strictly prohibited, and negative practices such as ostentatious comparisons or clique exclusion must be promptly addressed.

Vigilance is needed to prevent secondary harm from excessive screening or labeling, and psychological services should remain voluntary and confidential. Value guidance must avoid hollow slogans and integrate with practical work contexts. Consistent leadership modeling is essential, though altering entrenched informal norms and cultural climates demands sustained commitment and high-level support.

(2) Establish institutionalized and diversified communication networks and social support platforms.

To mitigate isolation from social comparison and enhance organizational integration, structured communication mechanisms must be mandatorily implemented. First, a mentorship program pairing new rural-origin civil servants with experienced non-rural-origin senior staff possessing strong communication skills should be formalized. Mentors must provide at least 1 year of job guidance, resource navigation, and psychological support, with mentorship outcomes incorporated into their performance evaluations. Second, regular structured leader-staff dialogues with fixed agendas should be institutionalized, ensuring equal opportunity for rural-origin civil servants to directly voice work experiences, challenges, and career aspirations to leadership. Responses must be provided, and issue-tracking systems maintained. Third, non-work affinity groups based on shared interests should be organized and funded by unions or party-mass departments, encouraging cross-departmental and cross-background participation to reduce comparison and strengthen collegial bonds.

Potential pitfalls include mentorships exacerbating divides if mismatched or superficial, dialogues becoming performative without leadership preparation and feedback mechanisms, and activities imposing burdens if poorly designed or mandatory. Communication barriers arising from generational or positional differences must also be addressed.

(3) Design transparent and development-oriented incentive mechanisms and promotion pathways to strengthen social mobility beliefs.

Addressing the moderating role of social mobility beliefs requires ensuring rural-origin civil servants clearly perceive the positive link between effort and reward and upward mobility potential. First, performance evaluation, recognition, and promotion criteria, processes, and outcomes must be comprehensively standardized, transparently disclosed, and made verifiable, with accessible channels for queries and appeals. Perceptions of “hidden barriers” or “favoritism-based advancement” must be eliminated. Second, alongside regular promotions, specialized initiatives (e.g., “Grassroots Talent Development Programs” or “Capability Advancement Projects”) should provide additional training, critical task assignments, or cross-departmental rotations for high-potential rural-origin civil servants potentially constrained by background. Participation outcomes should form documented “growth portfolios” prioritized in promotion decisions. Third, quantifiable performance contributions must be significantly weighted in material incentives, supplemented by non-monetary honors like “Dedication Awards” or “Innovation Breakthrough Awards” to enhance achievement recognition and organizational belonging.

Special initiatives risk being perceived as “patronage” or creating new relative deprivation among others if poorly designed or communicated, necessitating an emphasis on merit- and potential-based selection. Transparency may expose existing systemic inequities, requiring courage for concomitant reforms. Fairness in material incentives depends heavily on scientifically objective performance evaluation systems, presenting inherent management challenges. Sustained resource commitment is vital to ensure training and development effectiveness.

(4) Implement dynamic monitoring and evaluation feedback mechanisms to ensure policy implementation and optimization.

A closed-loop monitoring and evaluation system is essential to prevent superficial implementation or unintended consequences. First, “work engagement and organizational integration of rural-origin civil servants” must be incorporated as specialized metrics in annual organizational health assessments, tracked continuously via anonymous surveys, structured interviews, and behavioral observation to monitor changes in work withdrawal, psychological depletion, social comparison frequency, and social mobility beliefs. Second, “Organizational Health Observer” groups comprising diverse employees should be established to regularly collect frontline feedback and assess policy effectiveness and potential issues. Third, based on monitoring data and feedback, specialized reviews led by higher-level organizational departments must be convened periodically, bringing together experts, management representatives, and rural-origin civil servant delegates to diagnose problems, evaluate policy efficacy, and dynamically refine intervention strategies.

Resource investment is required to ensure monitoring rigor and data authenticity. Observer mechanisms need adequate authority and protection to avoid tokenism or apprehension. Policy adjustments must balance stability and flexibility to prevent inconsistency. Deep-seated structural issues revealed may necessitate higher-level systemic reforms.

### 5.3 Limitations and future directions

#### 5.3.1 Sample scope and representativeness

The sample primarily focused on rural-origin civil servants with 10 years or less of work experience within Jinan City. Future research could expand the sample to include civil servants across diverse geographic regions (e.g., varying levels of economic development, different tiers of government) and with a broader range of work tenures. This will enhance the generalizability of the findings. Additionally, employing stricter random sampling techniques would further improve the representativeness of future studies.

#### 5.3.2 Research design and measurement

The cross-sectional nature of this study limits the ability to establish causal directionality among the variables. Future research could employ longitudinal designs or quasi-experimental methods to track changes over time and provide stronger evidence for causality. Furthermore, while adapted scales were used, future studies may benefit from more extensive validation of measurement instruments within this specific cultural and occupational context, potentially exploring context-sensitive indicators for constructs like behavioral depletion.

#### 5.3.3 Cultural contextualization

The study was conducted within the specific context of the Chinese civil service system. Future research could explore the generalizability of these findings to other cultural settings or occupational groups. A deeper integration of culturally specific factors (e.g., collectivism, Confucian values, guanxi) into the theoretical model could provide richer insights into the psychological mechanisms at play.

### 5.4 Conclusions

This study examines psychological and behavioral challenges arising from identity disparity among rural-origin civil servants in the workplace. Through empirical analysis of survey data from 1,137 rural-origin civil servants in Jinan, Shandong Province, the mechanism by which upward social comparison influences work withdrawal behavior was systematically investigated. A comprehensive theoretical model was proposed and validated, incorporating ego depletion—comprising cognitive, emotional, and behavioral dimensions—as the core mediating variable, with social mobility belief identified as the critical moderating variable.

## Data Availability

The raw data supporting the conclusions of this article will be made available by the authors, without undue reservation.
